# Selection of Candidate Monoclonal Antibodies for Therapy of Botulinum Toxin Type A Intoxications

**DOI:** 10.3390/toxins16070284

**Published:** 2024-06-21

**Authors:** Natalia A. Zeninskaya, Alena K. Ryabko, Maksim A. Marin, Tatyana I. Kombarova, Maria A. Shkuratova, Methun M. Rogozin, Marina V. Silkina, Yana O. Romanenko, Tatiana A. Ivashchenko, Igor G. Shemyakin, Victoria V. Firstova

**Affiliations:** 1 Laboratory of Molecular Biology, Federal Budget Institution of Science «State Research Center for Applied Microbiology and Biotechnology», Territory “Kvartal A”, 24, Obolensk, u.d., 142279 Serpukhov, Moscow Region, Russia; 2 Laboratory of Biological Trials, Federal Budget Institution of Science «State Research Center for Applied Microbiology and Biotechnology», Territory “Kvartal A”, 24, Obolensk, u.d., 142279 Serpukhov, Moscow Region, Russia

**Keywords:** botulism, monoclonal antibodies, hybridoma, mouse bioassay

## Abstract

Botulism is one of the most serious food intoxications, manifesting as prolonged paralytic conditions. This disease is usually the result of the consumption of poor quality canned or smoked foods, so the inhabitants of many countries of the world are exposed to the risk of this kind of poisoning every year. In view of the severity of poisonings caused by botulinum neurotoxins, monoclonal antibodies (mAbs) show great promise because of their targeting action, lack of allergic reactions and serum sickness. The use of a cocktail of mAbs increases the “functional specificity” of their mixture, allowing them to bind to the active domains of different toxin chains and block their action. In this work, we obtained 14 murine mAbs to the catalytic and receptor-binding domain of botulinum toxin type A. The Sp2/0-Ag14 murine myeloma cell line and splenocytes from immunized mice of the BALB/c line were used as fusion partners. We have shown that the selected cocktail of three antibodies neutralizes native toxin more effectively than antibodies separately—complete neutralization is achieved at a toxin dose of 3LD50 and partial neutralization at 5LD50. We presume that this cocktail may be promising as a prototype for the creation of a therapeutic drug capable of neutralizing the toxin in the blood of patients.

## 1. Introduction

Botulinum toxin, synthesized by the anaerobic spore-forming soil bacterium *Clostridium botulinum* and less commonly by other representatives of the genus *Clostridium*, is the most dangerous of the natural poisons. There are eight types of botulinum toxins (A-H) and a variety of subtypes. The clinically significant ones causing severe food intoxication in humans are type A, B and E toxins [1,2,3]. All botulinum toxins are binary in nature and consist of a light chain and a heavy chain. The heavy chain is represented by a receptor-binding domain and a translocation domain. The light chain is a catalytic domain and a zinc endopeptidase. Depending on the type of toxin, it cuts different proteins of the soluble N-ethylmaleimide sensitive factor attachment protein receptor (SNARE) family (synaptosomal associated protein (SNAP-25), synaptobrevin (VAMP) and syntaxin), preventing the exocytosis of acetylcholine from the neuron, which leads to muscle paralysis. For effective therapy, the antidote must inhibit the action of one or more domains of the toxin by blocking their functionality [4,5,6].

In most cases, botulism is presented in the form of severe food intoxication; forms of neonatal botulism and wound botulism are much less common. The prognosis of this disease depends on the early initiation of treatment and the severity of the course. Therefore, special attention should be paid to early diagnosis and rapid administration of an antitoxic drug. Currently, equine antitoxic sera containing polyclonal antibodies (pAbs) against 2–7 types of toxin are widely used. Their intravenous administration allows neutralization of the toxin remaining in the bloodstream but, of course, does not allow reversal of the action of the toxin in neurons that have already cut the substrate. In addition, this type of therapy leads to the development of serum sickness and allergic reactions. In contrast to pAbs, mAbs, having a more specific and unidirectional action, can be produced faster, in much larger volumes and without batch-to-batch variations [1,7,8].

This work is focused on obtaining the most effective combination of mAbs to botulinum toxin type A, which can serve as a prototype for the creation of a treatment against botulinum intoxication. In the future, this research will involve the chimerization of the resulting mAbs, as well as the development of a cocktail of antibodies against type A, B and E toxins based on them.

## 2. Results

### 2.1. Specific Activity of Antibodies against Closely Related Molecules

**Figure 1:**
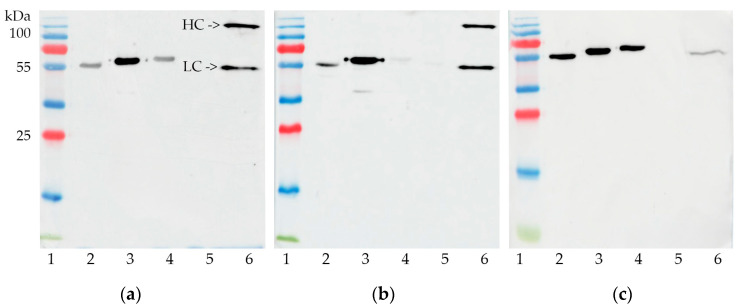
Immunoblot of botulinum antigens against mAbs CB-LCA_1-4 (**a**), CB-HCA_2-11 (**b**), CB-LCA_2-55 (**c**): 1—PageRuler™ Plus Prestained Protein Ladder (Fermentas, USA), 2—rBoNT/A-HC50, 3—rBoNT/A-LC, 4—rBoNT/B-HC50, 5—rBoNT/B-LC, 6—native BoNT/A.

During the selection stages, we managed to obtain 13 stable hybridomas synthesizing mAbs to rBoNT/A-LC and 1 to rBoNT/A-HC50. These mAbs exhibit specific activity not only against the target protein and BoNT/A, but also in most cases against other considered antigens, except rBoNT/B-LC (Figure 1, Table 1). We saw a similar relationship when analyzing antibodies to BoNT/A produced by human/mouse heterohybridomas, which were obtained in our laboratory (data not published). According to the literature data, botulinum toxins have identity between types (32–64% amino acid sequence identity), as well as some sequence homology with tetanus toxin [9]. The differences between toxins within the type are not so significant; however, even a difference of 10% can affect the affinity of mAbs to the antigen, changing them by several orders of magnitude [10,11,12]. This variability may affect the binding of mAbs to different types and subtypes of botulinum toxins, but it is unpredictable and very situational. No one has previously made identity comparisons between botulinum toxin domains—after all, these are actually separate functional units. We compared the sequences of our recombinant proteolytic and receptor-binding domains of BoNT/A and BoNT/B by VectorBuilder’s Sequence Alignment tool. The protein identity ranged from 20.64 to 41.86%.

We assume that the proteolytic and receptor-binding domains of different types of botulinum toxins can form similar conformational epitopes, which in general can be useful in our future developments when selecting cocktails to neutralize botulinum toxins A, B and E. However, taking into account the values of the identity indicators, we consider this similarity to be coincidental.

### 2.2. Inhibitory Activity of Antibodies against the Proteolytic Action of the Light Chain of Botulinum Toxin Type A

The FRET-assay is a very sensitive method; therefore, to obtain reproducible results, it is very important to strictly observe the molarity of the working buffer components as well as the storage conditions of the fluorogenic substrate; otherwise, the obtained data may change dramatically. BoNT/A light chain is a zinc-dependent endopeptidase. Accordingly, in the absence of a sufficient amount of ZnSO_4_, the activity of this enzyme decreases. The optimal ratio of the FRET substrate, protease and antibodies is selected experimentally [13]. It is recommended that a series of measurements be taken to monitor the growth and shape of the exponential curve of RFU values. This will help to verify whether the experiment was set up correctly.

**Figure 2:**
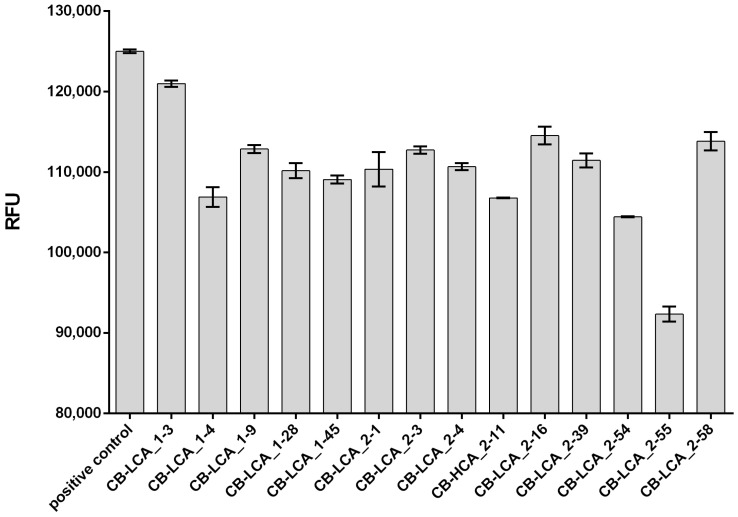
The ability of antibodies to inhibit the proteolytic activity of the light chain of botulinum toxin type A. The RFU value is presented as Mean + SD at 4 h after the start of the reaction. The positive control is presented as rBoNT/A-LC and SNAPtide in working buffer solution. Each value is blanked against the negative control (SNAPtide in working buffer solution).

The mAb CB-LCA_2-55 exhibits maximum inhibitory activity under these conditions (Figure 2). Among the remaining antibodies, there is some correlation between their inhibitory activity, as well as specific and protective activity in vivo, but it is not so significant. An unexpected result was the ability of mAb CB-HCA_2-11 to influence the proteolytic activity of rBoNT/A-LC. This can only be explained by the fact that this antibody is also specific for this recombinant protein (Figure 1b).

### 2.3. Antitoxic Activity of Antibodies against Validated Native Toxin and Selection of the Most Effective Combinations of Antibodies by Mouse Bioassay

Analysis of toxin-neutralizing activity (TNA) was performed using a mouse bioassay, which is still the gold standard for the determination of botulinum toxins [14]. The drug Botox (Myotox) was used in this case as a ready-made validated toxin preparation and also to reduce the number of animals participating in this series of experiments [15]. The only serious disadvantage in using such a preparation is the inability to determine the subtype of toxin used. In this regard, the TNA could be lower.

**Figure 3:**
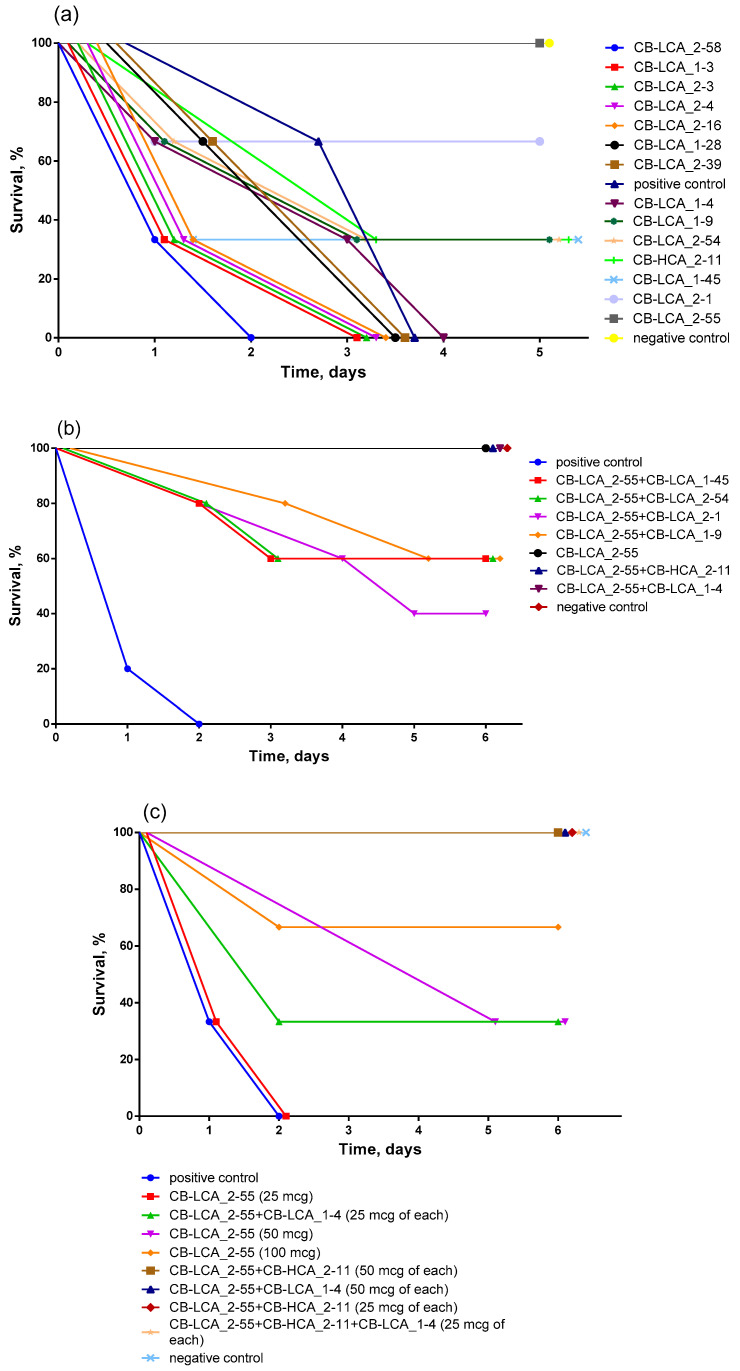

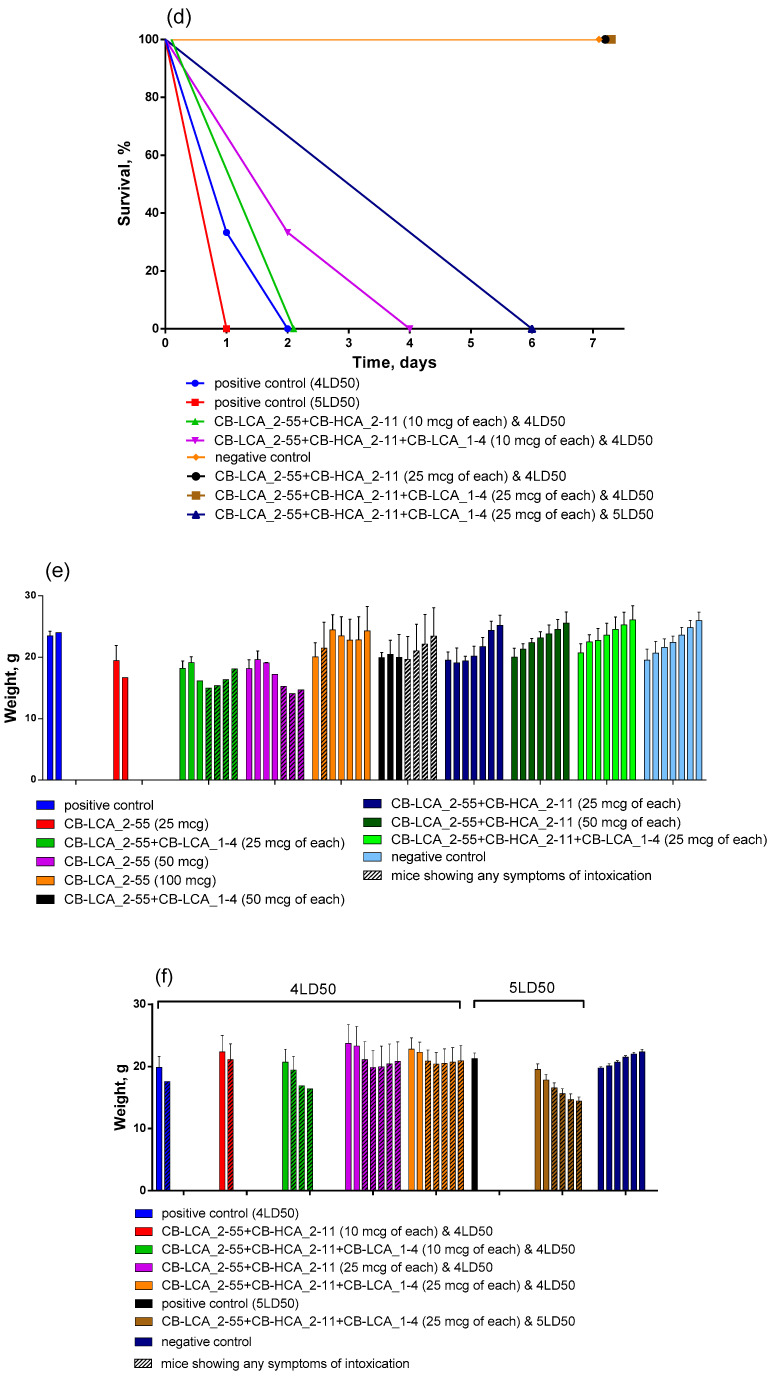
(**a**) Stage 1: survival of mice with a toxin load of 2LD50 and a mAb dose of 100 μg/mouse. N animals = 3; (**b**) Stage 2: survival of mice with a toxin load of 2LD50 and a mAb dose of 100 μg/mouse. The ratio of antibodies in mixtures by mass is 1:1. N animals = 5; (**c**) Stage 3: survival of mice with a toxin load of 3LD50 and mAb dose titration. The ratio of antibodies in mixtures by mass is 1:1. N animals = 3; (**d**) Stage 4: survival of mice with 4LD50 and 5LD50 toxin loading and mAb dose titration. The same ratio of antibodies in mixtures by weight is maintained. N animals = 3; (**e**) Stage 3: graph of mouse weight changes during 3LD50 toxin loading and mAb dose titration. The same ratio of antibodies in mixtures by weight is maintained. The weight value is expressed as Mean + SD for each individual day; (**f**) Stage 4: graph of mouse weight changes during 4LD50 and 5LD50 toxin loading and mAb dose titration. The same ratio of antibodies in mixtures by weight is maintained. The weight value is expressed as Mean + SD for each individual day.

The first stage involved the selection of mAbs that exhibit at least a minimal antitoxic effect. The 2LD50 dose was chosen to begin this series of experiments as it is close to full lethal. Eight antibodies were selected, the protective activity of which made it possible to at least minimally prolong the life of animals (Figure 3a). Among them, one antibody, CB-LCA_2-55, ensured the survival of the entire group of animals and served as the basis for further assembly of mAb combinations.

In the second stage, our goal was to make sure that combinations of our antibodies actually provide a more pronounced therapeutic effect than using them individually. According to the literature, cocktails of several protective antibodies to different domains of the toxin can enhance protection by several orders of magnitude due to the fact that they act on its different active centers [16,17,18]. As expected, all groups in the antibody pairs performed better than in the previous stage (Figure 3b). For the next step, only those mAbs were selected that, as part of the mixtures, ensured complete survival of mice in their groups (CB-LCA_1-4, CB-LCA_2-55, CB-HCA_2-11).

In final stages (3 and 4), the toxin dose was increased up to 3-5LD50, and the amount of mAbs used in pairs and triples was lowered (to 20 μg of total antibody mass per mouse) to determine the limits within which TNA was manifested. Due to the fact that with the increased efficacy of the resulting combinations, many animals initially felt good or were able to recover from the effects of the toxin during the course of the experiment, the animals’ well-being was monitored with daily weight measurements.

**Figure 4:**
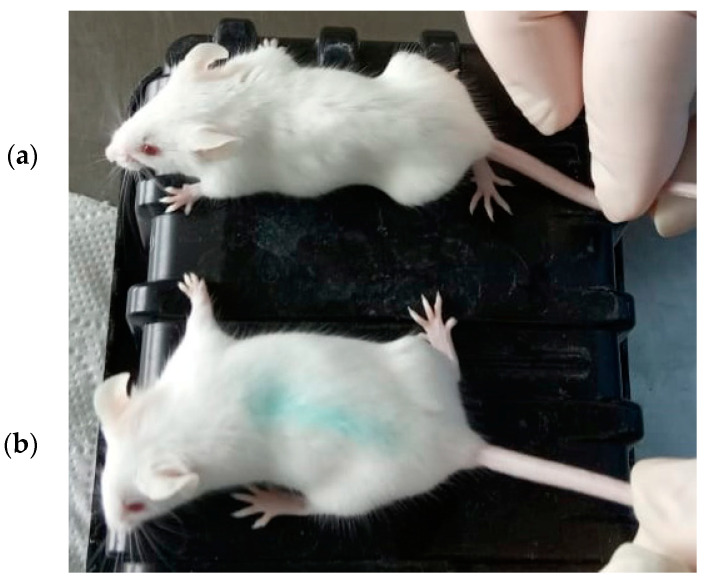
(**a**) Mouse exhibiting symptoms of botulinum toxin intoxication; (**b**) healthy mouse.

Observed clinical symptoms of illness and death of mice during botulinum intoxication are very characteristic (Figure 4b): the fur is ruffled; breathing is difficult; the muscles of the abdominal wall are weakened and sunken, giving the symptom of a “wasp waist”; and convulsions and paralysis of the hind limbs appear, followed by death due to complete paralysis due to respiratory failure [19,20].

According to the results of the experiment, the best antibody combination was CB-LCA_2-55 + CB-HCA_2-11 + CB-LCA_1-4 (at a dosage of 25 mcg of each mAb), which neutralized the effect of botulinum toxin type A with equal efficiency. At a dose of 3LD50, the negative effect of the toxin does not affect the mice in any way, and they gain weight similarly to animals in the negative control group (Figure 3c,e); at 4LD50, they are able to recover by the end of the experiment. However, the 5LD50 dose is the threshold dose when the effects of the toxin are neutralized, but the animals are not able to recover from the effects of its influence (Figure 3d,f).

### 2.4. Characterization of Selected Antibodies

**Figure 5:**
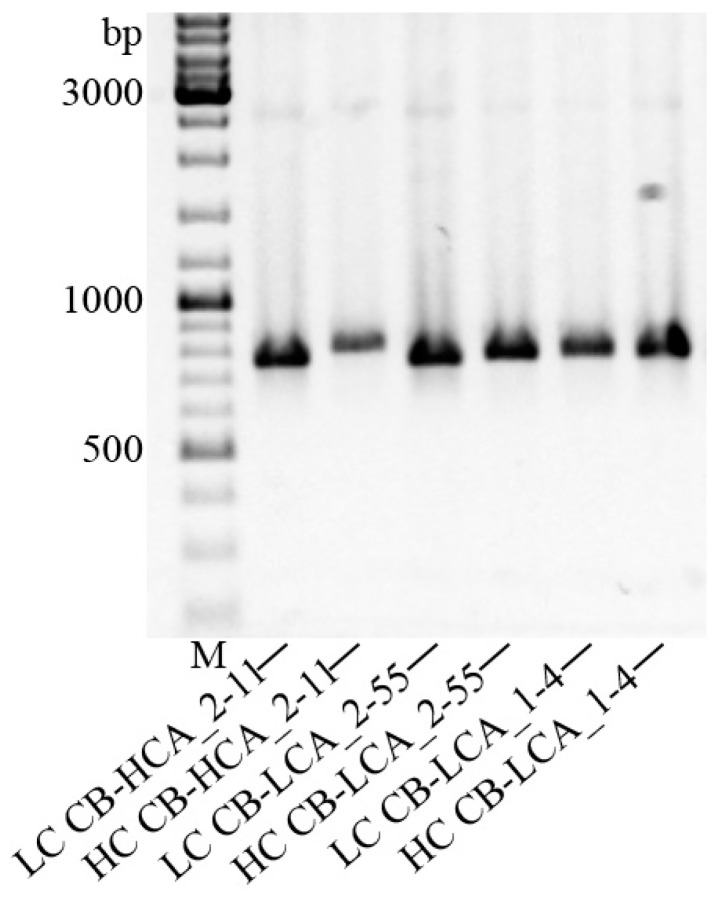
RACE-PCR of selected antibodies. Lanes were loaded with 2 μL of the reaction product. M—DNA molecular weight marker GeneRuler DNA Ladder Mix (Thermo Scientific, Waltham, MA, USA), LC—mAb light chain, HC—Fd region of mAb heavy chain.

Through analysis of antibody sequences, we were able to find out that all antibodies of interest have a G1 heavy chain type and a κ light chain type. The fragments of mAb light and heavy chains obtained during initial amplification are shown in Figure 5. The CB-HCA_2-11 antibody was found to have higher affinity towards its targeting protein (rBoNT/A-HC50) (Table 2). However, as we found out earlier, it does not affect neutralization of the toxin in a key way due to the fact that this mAb prevents the transport of LC BoNT/A inside the cell but not the cleavage of the SNAP-25 protein involved in acetylcholine transport.

## 3. Discussion

Botulism is one of the most dangerous food intoxications. Despite the rarity of this disease, the risks of poisoning are constant due to the peculiarities of the traditional diet of the population and sometimes frivolous attitude to the production and consumption of canned products and smoked meats, where the pathogen multiplies and synthesizes the toxin. The high lethality of this disease and long recovery period of patients underscore the importance of emergency therapy [21]. The exact number of cases worldwide is difficult to ascertain; there are thought to be 1000–2000 cases of foodborne botulism per year [22,23]. The therapeutic window for antitoxic therapy is approximately 24 h but technically cannot be determined due to the direct dependence of the effects of poisoning on the amount of toxin absorbed by the patient with food [21,24]. Therefore, any means of express diagnostics will be extremely useful.

In this work, we were able to obtain 14 mouse hybridomas synthesizing mAbs against BoNT/A. The obtained antibodies mostly showed specific activity when analyzed by immunoblotting against not only the target recombinant protein and native BoNT/A but also against the other analyzed proteins, except rBoNT/B-LC. We hypothesize that if similar cross-reactivity to several targeting molecules at once occurs during the next generations of hybridomas to other toxins, a more efficient cocktail of antibodies to several types of botulinum toxin at the same time may result.

MAbs cross-reactivity has been previously reported in many studies by Garcia-Rodriguez C et al. The analyzed single-chain fragment variable antibodies were active within one type of toxin (subtypes A1 and A2) to the variable epitope of the receptor-binding domain. The authors noted a 29,000-fold difference in antibody affinity between the two subtypes and a more than 1000-fold increase in activity to the A2 subtype after the modifications [12]. Also of interest is their other work [25], which examined multispecific human mAbs that exhibited activity against different types of botulinum toxins. This was possible due to the fact that the analyzed antibodies interacted with a highly conserved epitope of the translocation domain.

Certain mAbs block the catalytic activity of BoNT/A towards SNAP-25, which helps to neutralize the toxin. SNAPtide, a FRET substrate composed of a 13-amino acid peptide derived from SNAP-25, serves as a valuable tool for investigating the mechanism of action of BoNT/A and evaluating the efficacy of its potential inhibitors [26]. We initially studied the acquired mAbs for their ability to inhibit the cleavage of the SNAPtide substrate, employing rBoNT/A-LC as the enzyme source. It was established that all the antibodies exerted blocking effects to varying degrees on catalytic activity and should be further investigated in the animal model, with particular consideration given to the prospective mAb CB-LCA_2-55. However, it should be noted that other studies have observed that even protective mAbs F1-2 and F1-40 can enhance the endopeptidase activity of the light chain of BoNT/A and BoNT/A holotoxin in the SNAPtide assay [27]. There are neutralizing antibodies against BoNT/A that block the intracellular cleavage of SNAP-25 but do not disrupt the catalytic activity of the light chain, so the FRET-assay is an initial and valuable addition to studying new mAbs.

In our work, we experimentally selected 3 antibodies out of 14 which act much more effectively in a cocktail than separately. The selection of an antibody cocktail was motivated by earlier studies, for example, Nowakowski A et al. [17], who showed that individual antibodies have a weaker protective effect than a combination of oligoclonal mAbs to non-overlapping epitopes of the receptor-binding domain. In another work, Diamant E. et al. [28] presented multispecific oligoclonal mAbs against botulinum toxins A, B and E. When combining serotype-specific monoclonal antibodies, a strong synergistic effect was obtained, consisting of a 400-fold increase in neutralizing activity. The antibody composition of CB-LCA_2-55, CB-HCA_2-11 and CB-LCA_1-4 completely protects mice at a toxin dose of 3LD50 and provides partial neutralization with an extension of the life of animals at a dose of 5LD50 BoNT/A. It is possible that this level could have been higher if our substance of the toxin (Myotox) did not correspond to subtype A1, against which the antibodies were generated.

The use of murine monoclonal antibodies in therapy may have a number of disadvantages similar to the use of equine antitoxic serum. However, it is known that chimerization of antibodies often improves their efficacy, increases their half-life and also minimizes possible immunogenicity due to the replacement of most of the mouse regions with human counterparts [29]. We obtained cDNA sequence data for antibodies CB-LCA_2-55, CB-HCA_2-11 and CB-LCA_1-4 and will design recombinant chimeric antibodies based on them in our upcoming work. It would be beneficial to determine whether the protective effect after antibody chimerization remains or increases.

## 4. Conclusions

Thus, we obtained murine mAbs against botulinum toxin type A. We also managed to select a combination of these antibodies that effectively protects mice up to a 5LD50 dose of the toxin. This is just the first step of the study, but in the future, a drug based on a cocktail of chimeric mAbs to botulinum toxins A, B and E can become a full replacement for the current clinical practice of using equine antitoxic serum in view of minimizing adverse reactions, as well as a more specific and targeted effect.

## 5. Materials and Methods

### 5.1. Ethics Statement

All animal experiments were carried out in full compliance with the European Convention for the Protection of Vertebrate Animals Used for Experimental and Other Scientific Purposes (Directive 2010/63/EU of the European Parliament and of the Council of 22 September 2010 on the protection of animals used for scientific purposes), as well as the requirements of Russian Federation Sanitary Rules 1.3.2322-08 “Safety of working with microorganisms of III–IV groups of pathogenicity and pathogens of parasitic diseases” and the Veterinary Protocol on Bioethics of the FBIS SRCAMB #VP-2024/1 dated 30 January 2024.

### 5.2. Production of Recombinant Proteins

Synthetic DNAs encoding proteins of the light chain and C-terminal receptor-binding domain of the heavy chain of botulinum toxin types A and B (rBoNT/A-LC, rBoNT/B-LC, rBoNT/A-HC50 and rBoNT/B-HC50) of Clostridium botulinum were cloned into plasmids of the pET family (Novagen, Madison, WI, USA) in a manner similar to that described in [30]. The resulting recombinant plasmids were used to produce proteins in Escherichia coli strain BL21(DE3) in the presence of 1 mM IPTG at 30 °C. Proteins were isolated from the cell lysate by metal-chelate chromatography on a Tricorn 10/50 column (GE Healthcare, Chalfont Saint Giles, UK) with TALON resin (Takara BIO, San Jose, CA, USA) followed by size exclusion on a Superdex-200 10/300 GL column (GE Healthcare, UK) [31].

### 5.3. Preparation of Botulinum Toxin Type A

The toxin was obtained according to the method described by Malizio [32]. *Clostridium botulinum* ATCC 13124 (NCIM FBIS SRCAMB, Obolensk, Russia) was used as a strain-producer. After precipitation and extraction, the suspension with isolated toxin was stored in ammonium sulfate solution with 60% saturation at +4 °C.

The obtained toxin, due to its instability in pure form, was used only in immunoblotting. This toxin belongs to subtype A1.

### 5.4. Immunization of Animals

Eight-week-old male BALB/c line mice were immunized according to the classical two-month protocol [33] with complete and incomplete Freund’s adjuvant in a 1:1 volume ratio with recombinant rBoNT/A-LC and rBoNT/A-HC50 proteins at a quantity of 100 µg/mouse. Injections were performed at 2-week intervals.

### 5.5. Hybridoma Production

Sp2/0-Ag14 murine myeloma cells and lymphocytes from immunized mice were isolated from spleens and lymph nodes, separated in a Ficoll gradient (Ficoll-Paque PLUS (GE Healthcare, UK)) and served as fusion partners. The fusion was carried out at a ratio of 1:5 using the electrofusion method, the protocol of which was described by us earlier [34].

The cells were further cultured in DMEM medium with 10% FBS supplemented with 1× GlutaMAX, 1× antibiotic–antimycotic and pressurized with 1× HAT. The feeder layer preparation step as well as the conversion from HAT to HT was excluded from the primary culturing. The selection period with HAT was 3 weeks. After screening for target proteins, the selected hybridoma clones were cloned twice and then cryopreserved.

### 5.6. Obtaining Antibodies from Ascitic Fluid

Young, six- to eight-week-old mice were injected with 500 μL of pristane intraperitoneally. After 2–3 weeks, implantation of hybridoma cell suspension in PBS at a quantity of 2–3 × 10^6^ cells/mouse was performed. Ascites fluid was collected after 1–2 weeks based on the condition of the animals. MAbs were purified by affinity chromatography on a Protein G Sepharose 4 Fast Flow resin (GE Healthcare, UK), followed by size exclusion chromatography on a Superdex 200 column (GE Healthcare, UK).

### 5.7. Analysis of mAbs Specificity by Immunoblotting

Antibody specificity was assessed by chemiluminescent Western blotting. Recombinant proteins rBoNT/A-LC, rBoNT/A-HC50, rBoNT/B-LC and rBoNT/B-HC50 (0.2 μg of protein per well) and native botulinum toxin type A (15 μL of suspension per well) were separated by PAGE electrophoresis in a 12% acrylamide gel under denaturing conditions. Replicas were transferred onto Hybond ECL nitrocellulose paper (GE Healthcare, Chicago, IL, USA) using a Mini Trans-Blot Cell wet blotting module (Bio-Rad, Hercules, CA, USA). Development was carried out using the SuperSignal West Dura Extended Duration Substrate Kit (Thermo Scientific, USA).

### 5.8. Determination of the Antibodies Ability to Inhibit Proteolytic Activity of the Light Chain of Botulinum Toxin Type A

The ability of mAbs to inhibit the proteolytic activity of recombinant rBoNT/A-LC protein was tested by the FRET-assay using commercial FRET substrate SNAPtide Botulinum Toxin A Substrate, Fluorogenic (Sigma-Aldrich, St. Louis, MO, USA) according to the manufacturer’s instructions on an EnSpire analyzer (Perkin Elmer, Waltham, MA, USA) in OptiPlate-96 plates (Perkin Elmer, USA). The working buffer solution included 20 mM HEPES pH 8.0, 5 mM DTT, 0.3 mM ZnSO_4_ and 0.1% Tween-20. The reaction was performed in an OptiPlate-96 96-well plate (Perkin Elmer, USA). The working concentration of the FRET substrate in the well was 5 μM, and 0.75 μM of rBoNT/A-LC protease was added along with 1.6 μM of mAb. Antibodies with protease were incubated on a thermostatic shaker at 37 °C for 60 min. FRET substrate solution was added to the wells immediately before placing the plate into the analyzer. The fluorescent signal was read over a period of 5 h at Ex/Em = 320 nm/420 nm. Each sample was prepared in triplicates.

### 5.9. Analysis of the Antitoxic Activity of mAbs Using a Mouse Bioassay

TNA analysis was carried out in several stages. Each series of experiments involved outbred female mice (18–24 g), with 3–5 animals per group (breeding facility of FBIS SRCAMB, Obolensk, Russia). To ensure reproducibility of the obtained data, a commercial drug Myotox (FSASI “M.P. Chumakov FSCRDIBP of RAS” (Institute of Poliomyelitis), Moscow, Russia) containing 100 units/mL of botulinum toxin type A with hemagglutinin, of the same production batch, was used. Each experiment involved selecting a toxin dosage (from 2 to 5LD50, where LD50 is 1 unit) and a mixture of monoclonal antibodies (not exceeding 100 μg), which has a toxin-neutralizing effect. Mice were intraperitoneally injected with a mixture of one or more antibodies and botulinum toxin in a total volume of 150 μL. Each stage of the experiment lasted at least 5 days and was completed 24 h after the last animal died. In the last stages, weighing and monitoring of the well-being of the animals was carried out in order to evaluate in more detail the effectiveness of the protection of the studied combinations of mAbs.

### 5.10. Determination of Isotypes

The cDNA fragments of the light and heavy chains of antibodies were obtained by the 5′ RACE-PCR method [35]. The obtained fragments were cloned into plasmid pUC19. Antibody isotypes were identified by analyzing the sequencing results. The coding sequences of the CH1 domain and light chain were analyzed in the BLAST program to establish the isotypic identity of antibodies.

### 5.11. Determination of the Equilibrium Dissociation Constant Values

The affinity of the obtained mAbs for target molecules was measured using the surface plasmon resonance (SPR) method on a Biacore X100 instrument (Cytiva, Sweden) and was used to calculate the equilibrium dissociation constant (K_D_). The analysis was performed at 25 °C in HBS-EP buffer. The CM5 sensor chip was immobilized with recombinant protein (rBoNT/A-LC or rBoNT/A-HC50) using His Capture Kit type 2 (Cytiva, Sweden) according to the manufacturer’s instructions. MAbs were administered in a series of two-fold dilutions in the concentration range of 62.5–1000 nM at a flow rate of 30 μL/min. The chip was regenerated with a solution of 10 mM glycine-HCl, pH 1.8. K_D_ was calculated by normalization and sensogram analysis using the 1:1 Langmuir binding model with Biacore X100 Evaluation Software (version 2.0.2).

## Figures and Tables

**Table 1:** Screening results for specific activity of monoclonal antibodies (native conditions/denaturing conditions).

#	mAb	Analyzed Antigens				
		rBoNT/A-LC	rBoNT/A-HC50	rBoNT/B-LC	rBoNT/B-HC50	Native BoNT/A *
1	CB-LCA_1-3	+/+	+/+	−/−	+/+	+
2	CB-LCA_1-4	+/+	+/+	−/−	+/+	+
3	CB-LCA_1-9	+/+	−/−	−/−	−/−	+
4	CB-LCA_1-28	+/+	+/+	−/−	+/+	+
5	CB-LCA_1-45	+/+	+/+	−/−	+/+	+
6	CB-LCA_2-1	+/+	+/+	−/−	+/+	+
7	CB-LCA_2-3	+/+	+/+	−/−	+/+	+
8	CB-LCA_2-4	+/+	+/+	−/−	+/+	+
9	CB-HCA_2-11	+/+	+/+	−/−	+/−	+
10	CB-LCA_2-16	+/+	+/+	−/−	+/+	+
11	CB-LCA_2-39	+/+	+/+	−/−	+/−	+
12	CB-LCA_2-54	+/+	+/+	−/−	+/+	+
13	CB-LCA_2-55	+/+	+/+	−/−	+/+	+
14	CB-LCA_2-58	+/+	+/+	−/−	+/+	+

*—sample of native BoNT/A protein is presented only under denaturing conditions.

**Table 2:** Characteristics of selected antibodies (dissociation constants and isotypes).

mAb	K_D_, M (to Target Protein)	Heavy Chain Type	Light Chain Type
CB-LCA_1-4	5.29·10^−7^	G1	κ
CB-HCA_2-11	8.97·10^−8^	G1	κ
CB-LCA_2-55	4.22·10^−7^	G1	κ

## Data Availability

Data are provided in the article.
